# Geometric ring aortic valve repair in tetralogy of Fallot: First reported case

**DOI:** 10.1016/j.xjtc.2024.03.016

**Published:** 2024-04-02

**Authors:** Marco Pocar, Giacomo Maraschioni, Cristina Barbero, Stefano Salizzoni, Alessandro Vairo, Carlo Pace Napoleone, Mauro Rinaldi

**Affiliations:** a Division of Cardiac Surgery, “Città della Salute e della Scienza” and Department of Surgical Sciences, University of Turin, Turin, Italy; b Department of Clinical Sciences and Community Health, University of Milan, Milan, Italy; c Division of Cardiology, “Città della Salute e della Scienza,” Turin, Italy; d Division of Pediatric and Congenital Cardiac Surgery, “Città della Salute e della Scienza”, Turin, Italy

**Figure:**
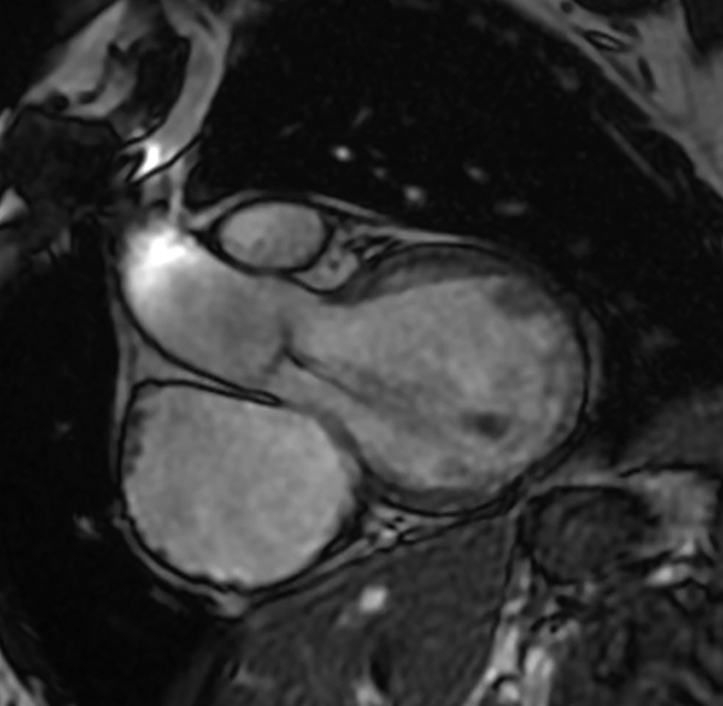
Aortic root dilatation and valve regurgitation late after TOF repair.

**Central Message**

Aortic root dilation and regurgitation are common in grown-up TOF. Reparative techniques rather than valve replacement may expand and anticipate surgical indications in this scenario.

Geometric ring annuloplasty is a recent adjunct in valve repair for aortic regurgitation (AR).^1^ This concept has sparingly been applied to congenital aortic or neoaortic valve dysfunction.^2^^,^^3^ Root dilatation is common in tetralogy of Fallot (TOF), particularly in adulthood late after repair, although severe AR and other complications are less frequent.^4^^,^^5^ We present the technical details of aortic valve repair comprising geometric prosthetic ring implantation in an adult patient with surgically corrected TOF. Institutional Review Board approval was not required; the patient provided written informed consent for publication of study data.

## Technique

**Figure 1:**
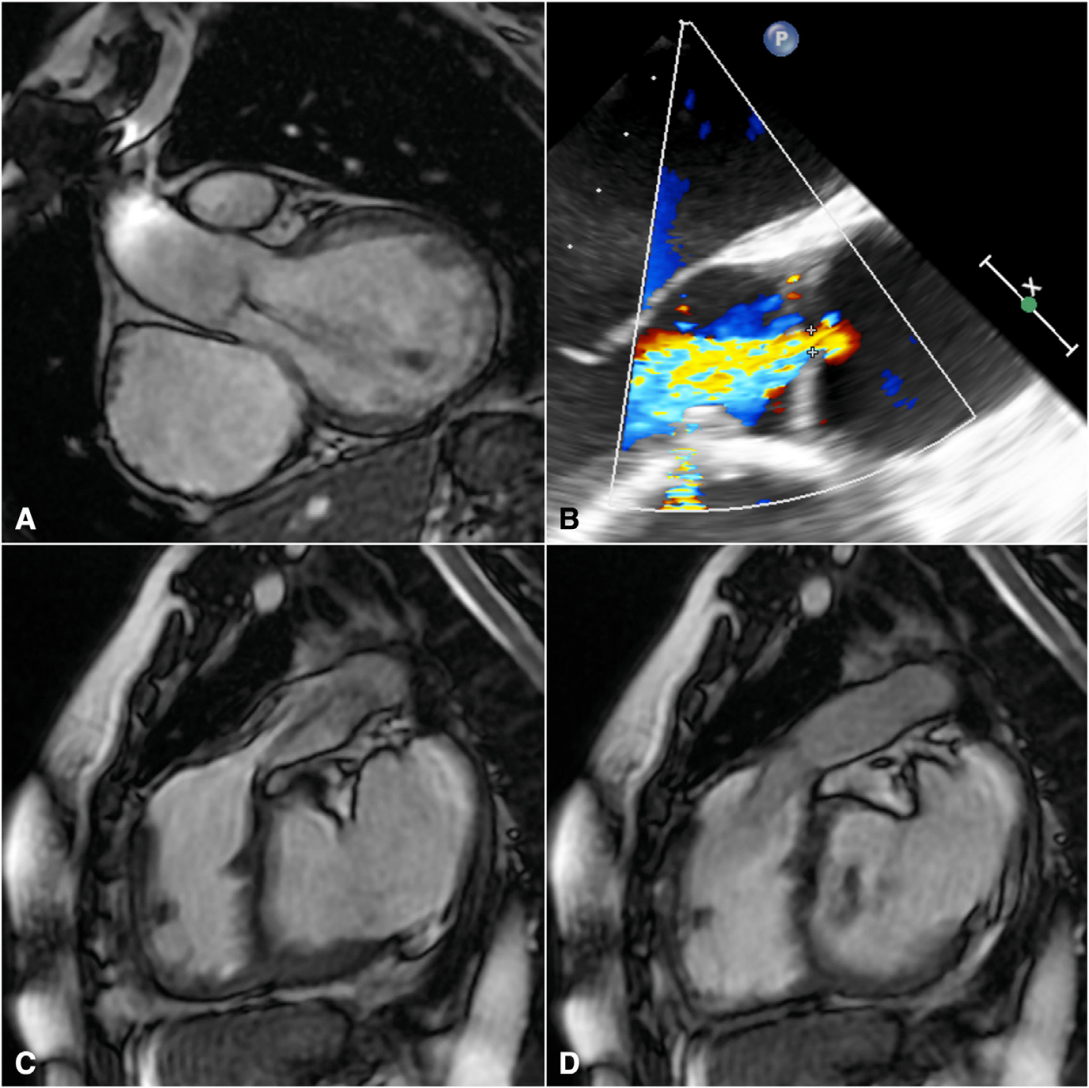
Preoperative AR shown by magnetic resonance imaging (A) and transesophageal echocardiography (B), and magnetic resonance depicting pulmonary homograft stenosis (C) and regurgitation (D).

A 47-year-old woman was referred for relapsing fever, pulmonary homograft structural degeneration with prevalent valve stenosis, and AR (Figure 1; Videos 1 and 2). She had initially undergone surgical correction of TOF with a transannular patch at 5 years of age and subsequent implantation of a pulmonary homograft and associated direct closure of a small residual ventricular septal defect 21 years thereafter. The sole associated anomaly was a right-sided aortic arch. Endocarditis was ruled out, and the patient was initially referred for pulmonary homograft and aortic valve replacement.

**Figure 2:**
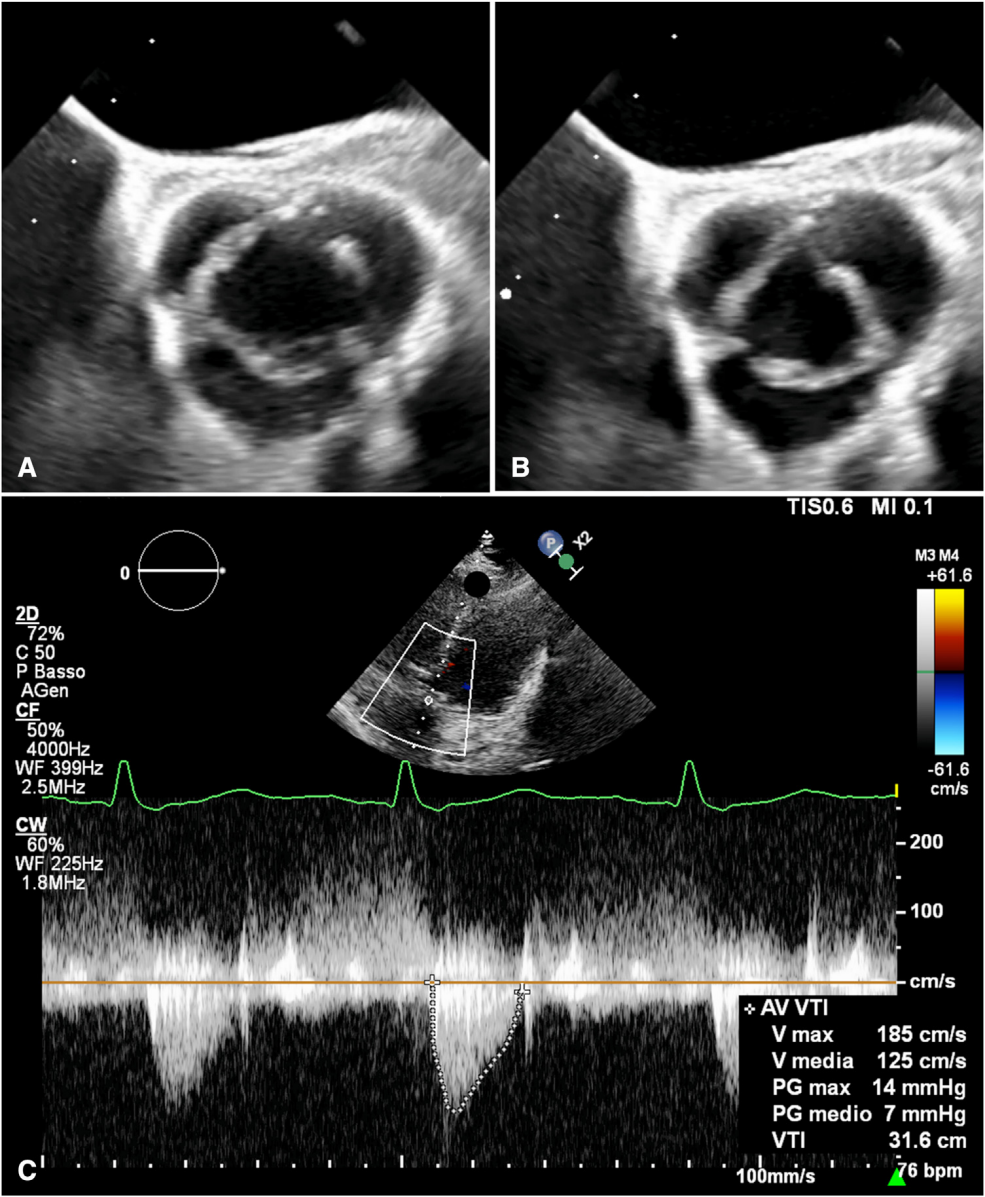
Systolic (A) and protodiastolic (B) short-axis appearance of the aortic valve after repair at 12-month transesophageal echocardiography. The shaved free margin of the noncoronary cusp (*upper left*) and central plication of the left coronary cusp (*upper right*) are evident. Transthoracic echocardiography confirms unobstructed valve opening with stable low postoperative gradients (C).

Transesophageal echocardiography revealed well-preserved aortic cusps and a predominantly central and near-symmetric regurgitant jet, whereas the aortic root maximal diameter measured 38 to 40 mm according to echocardiography and computed tomography, respectively. In consideration of young age and valve anatomy, the aortic valve was reconsidered for a conservative approach. Intraoperative assessment confirmed tricuspid morphology with a slightly diminutive noncoronary cusp coupled with mild-to-moderate prolapse of the left and minimal retraction of the right coronary cusps. The latter was most likely related to prior ventricular septal defect pericardial patch repair. An internal 21-mm HAART 300 geometric ring (BioStable Science and Engineering, Inc) was implanted and sized according to the free edge length and geometric height of the least represented noncoronary cusp.^1^ The latter was also shaved along the thickened free margin, whereas left cusp prolapse was corrected with a single interrupted 6-0 polypropylene central plication suture. The right cusp was left untouched. Implantation of a 26-mm pulmonary homograft with running 4-0 polypropylene sutures to replace the right ventricular outflow tract completed the operation. Transesophageal echocardiography documented trivial residual central AR, which remained stable at 12 months control, coupled with normalized ventricular dimensions (Figure 2; Videos 3-5). Peak and mean transvalvular gradients were 11 and 6 mm Hg, respectively.

## Discussion

Indications for valve repair in AR are steadily increasing in view of low mortality, fewer valve-related adverse events, and good survival. The main drawback remains recurrence and predictability of AR. In analogy to mitral repair, annular stabilization plays a key role to prevent AR recurrence. HAART rings were developed with this rationale, restoring physiologic geometry in tricuspid and, more recently, bicuspid aortic valves thereby abolishing the hazards of recurrent annular dilatation.

Geometric rings have been sparingly used to correct primary AR or neoaortic valve dysfunction in pediatric or grown-up congenital heart disease, with or without root remodeling. Valve pathology predominantly refers to autograft dysfunction after Ross procedures, followed by systemic valve regurgitation in patients with Fontan circulation or prior arterial switch operation, and truncal valve regurgitation after repair of truncus arteriosus.^2^^,^^3^ Aortic root ectasia is frequently observed late after TOF repair.^4^ However, despite intrinsic aortopathy and a root diameter often more than 4 cm, a study from the Mayo Clinic outlined low rates of progressive enlargement, significant AR, and acute aortic syndromes.^5^ Consequently, indications for reoperation on the aortic valve are sparingly reported. In this scenario, the mechanisms of AR are not primarily related to cusp involvement, but rather depend on annular dilatation. Thus, geometric ring annuloplasty appears an attractive option to treat AR in TOF anatomy. In the present case, the root was dilated with stable diameters (∼4 cm), sinotubular junction geometry was relatively well preserved, and wall thickness was normal. Thus, associated root remodeling was not indicated in view of the low likelihood of progressive aortic expansion.

## Conclusions

Although AR severity mandating reoperative surgery is seldom described, less severe AR with root ectasia is probably far more common in patients with TOF scheduled for reoperations. In relation to the different implicatioåns of ring implantation versus mechanical valve replacement, associated stabilization of the aortic annulus with a geometric ring may be considered also with a prophylactic attitude toward AR progression to prevent additional reoperations. Compared with other available techniques, namely, suture and external annuloplasty as widely reported by Drs Schäfers and Lansac, the choice was dictated by several factors. First, coronary dissection is unnecessary when buttons are not detached for root procedures, an undoubted advantage in reoperations. Although suture annuloplasty may be performed with less dissection, this may enhance distortion. Second, concerns have been raised in the pediatric population in relation to growth progressive shear stress leading to fibrosis at the hinge of the cusp,^6^ but this is not likely to pertain to adults. Third, a geometric ring intrinsically corrects annular asymmetry. Finally, the HAART ring is to date the sole suitable device for valve-in-ring transcatheter procedures. To the best of our knowledge, this approach has not been previously reported in TOF.

## Conflict of Interest Statement

The authors reported no conflicts of interest.

The *Journal* policy requires editors and reviewers to disclose conflicts of interest and to decline handling or reviewing manuscripts for which they may have a conflict of interest. The editors and reviewers of this article have no conflicts of interest.

## Supplementary Data

Video 1 Magnetic resonance showing moderately severe AR and moderate left ventricular systolic dysfunction (end-diastolic volume index, 138 mL/m^2^; ejection fraction, 52%). Video available at: https://www.jtcvs.org/article/S2666-2507(24)00145-7/fulltext.

Video 2 Structural degeneration of the pulmonary homograft with severe steno-insufficiency depicted by magnetic resonance imaging and secondary moderate right ventricular dysfunction (end-diastolic volume index, 106 mL/m^2^; ejection fraction, 46%). Video available at: https://www.jtcvs.org/article/S2666-2507(24)00145-7/fulltext.

Video 3 Intraoperative transesophageal echocardiography depicting absent residual regurgitation after discontinuation of cardiopulmonary bypass. Video available at: https://www.jtcvs.org/article/S2666-2507(24)00145-7/fulltext.

Video 4 Short- and long-axis color Doppler imaging at control transesophageal echocardiography. Video available at: https://www.jtcvs.org/article/S2666-2507(24)00145-7/fulltext.

Video 5 Short-axis view of the repaired aortic valve at control echocardiography. Video available at: https://www.jtcvs.org/article/S2666-2507(24)00145-7/fulltext.
