# Supplementary figures and images for: Geometric ring aortic valve repair in tetralogy of Fallot: First reported case
Source: JTCVS Tech. 2024 Apr 2;25:124–8. doi: 10.1016/j.xjtc.2024.03.016 (PMC11184622; doi:10.1016/j.xjtc.2024.03.016)

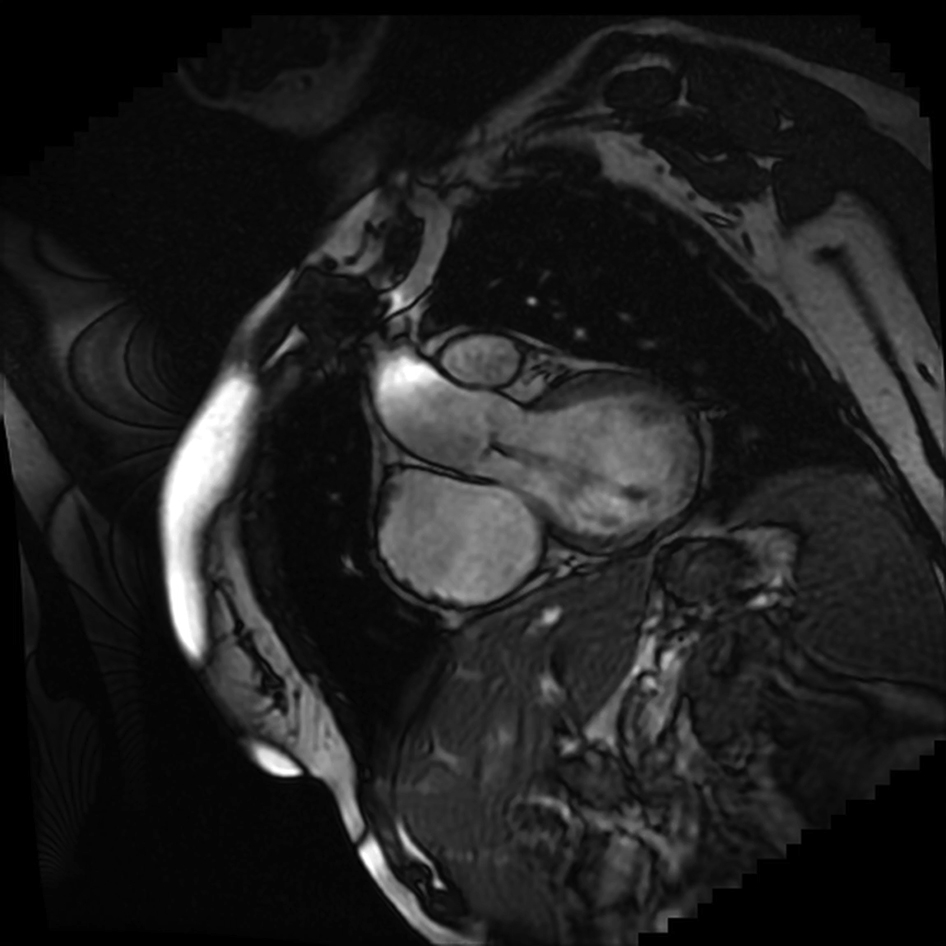

Supplement: Video 1 — Magnetic resonance showing moderately severe AR and moderate left ventricular systolic dysfunction (end-diastolic volume index, 138 mL/m2; ejection fraction, 52%). Video available at: https://www.jtcvs.org/article/S2666-2507(24)00145-7/fulltext. [file fx2.jpg]

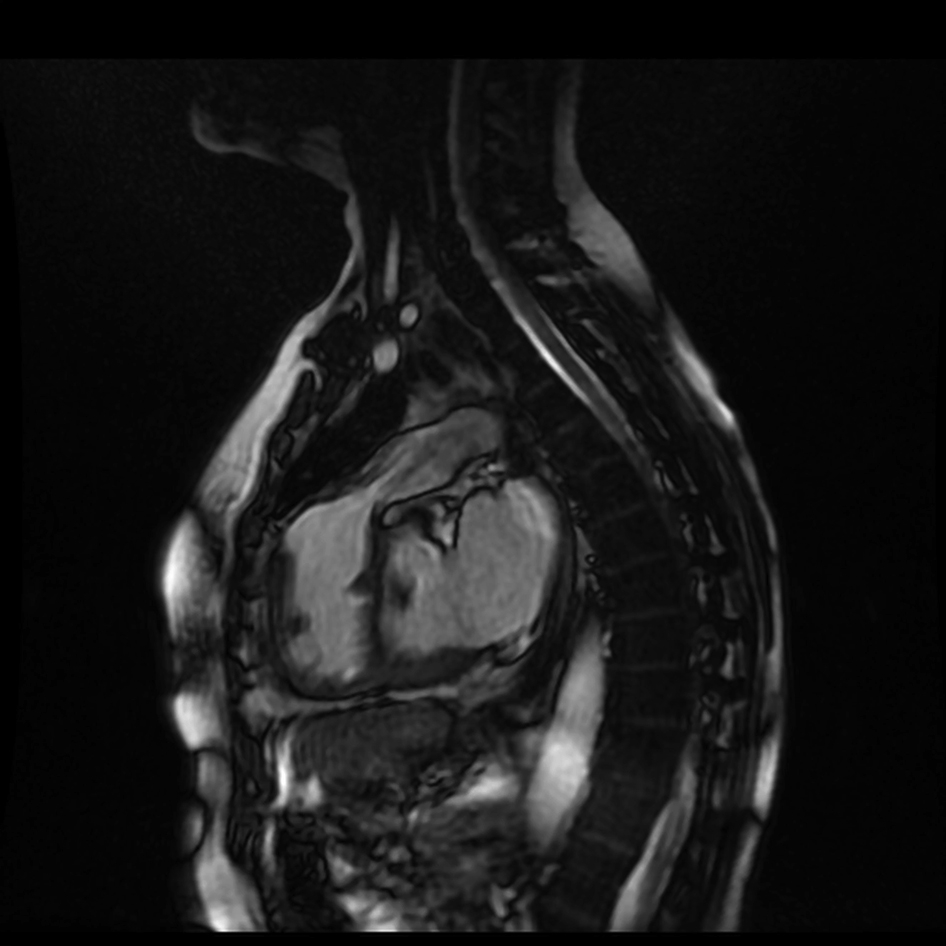

Supplement: Video 2 — Structural degeneration of the pulmonary homograft with severe steno-insufficiency depicted by magnetic resonance imaging and secondary moderate right ventricular dysfunction (end-diastolic volume index, 106 mL/m2; ejection fraction, 46%). Video available at: https://www.jtcvs.org/article/S2666-2507(24)00145-7/fulltext. [file fx3.jpg]

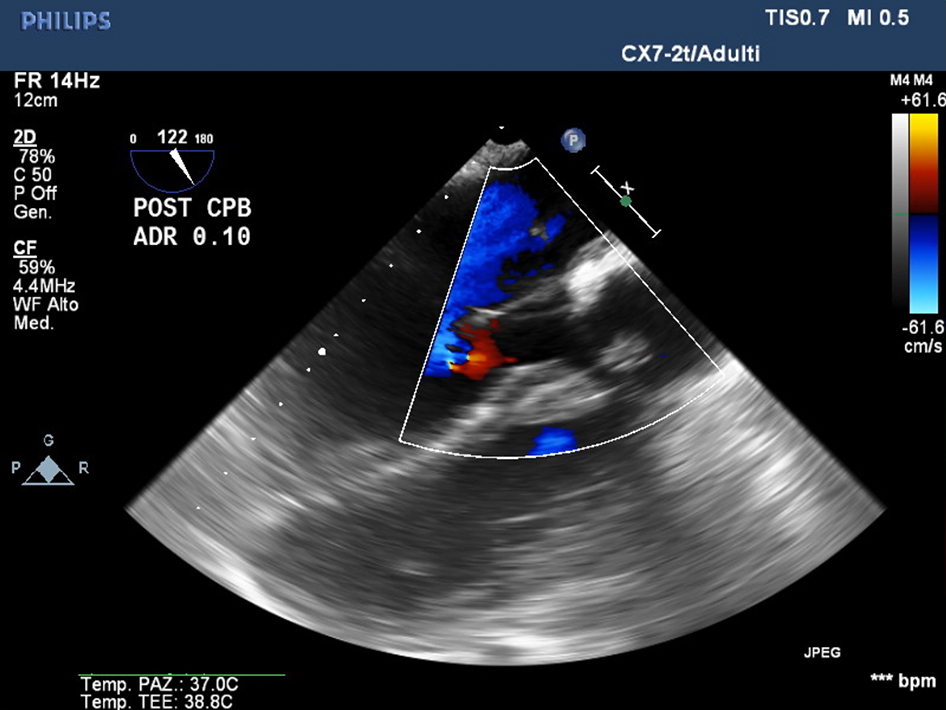

Supplement: Video 3 — Intraoperative transesophageal echocardiography depicting absent residual regurgitation after discontinuation of cardiopulmonary bypass. Video available at: https://www.jtcvs.org/article/S2666-2507(24)00145-7/fulltext. [file fx4.jpg]

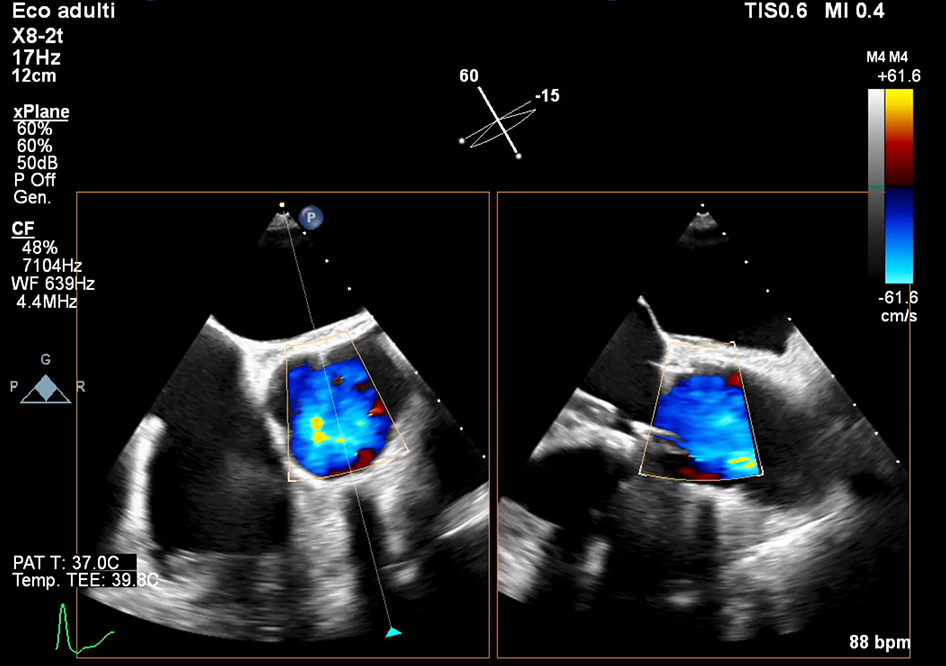

Supplement: Video 4 — Short- and long-axis color Doppler imaging at control transesophageal echocardiography. Video available at: https://www.jtcvs.org/article/S2666-2507(24)00145-7/fulltext. [file fx5.jpg]

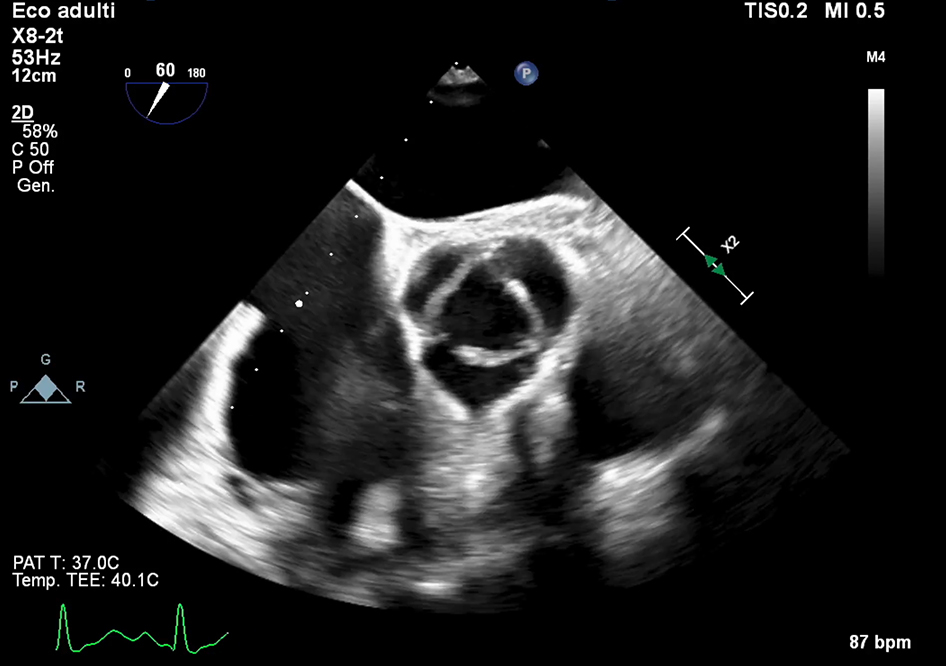

Supplement: Video 5 — Short-axis view of the repaired aortic valve at control echocardiography. Video available at: https://www.jtcvs.org/article/S2666-2507(24)00145-7/fulltext. [file fx6.jpg]
